# A novel hybrid approach for thyroid disease detection: Integrating cuttlefish algorithm and simulated annealing for optimal feature selection

**DOI:** 10.1016/j.mex.2025.103558

**Published:** 2025-08-07

**Authors:** Kapil Shrivastava, Saroj Pandey, Rishav Dubey, Mayank Namdev, Vipin Tiwari, Aditi Sharma

**Affiliations:** a Department of computer engineering and applications, GLA University, Mathura, Uttar Pradesh India; b Department of Computer Science and Engineering, Manipal University, Jaipur, Rajasthan, 303007, India; c Department of Computer Science and Engineering, Symbiosis Institute of Technology, Symbiosis International (Deemed University), Pune, 412115, India

**Keywords:** Cuttlefish, Simulated annealing, Machine learning, Feature selection, Thyroid disease, Hybrid approach

## Abstract

Effective treatment relies on a timely diagnosis, which is critical in the case of thyroid disorder—one of the chronic endocrine disorders alongside diabetes and obesity—with profound health concerns. Thyroid disorders occur due to the malfunctioning of the thyroid gland, which may result in an imbalanced metabolic rate due to inappropriate hormone levels synthesis. An overactive gland results in hyperthyroidism, whereas an underactive or sluggish thyroid lead to hypothyroidism. Both disorders, if not detected and managed timely, can lead to severe health complications. Early identification is crucial to delay or avoid debilitating complications and achieve a better quality of life through the right medical interventions and precise hormonal readjustments. The proposed hybrid algorithm method finds the best features for finding thyroid disease uses performance measures such as accuracy, F1-score, precision, and recall. The research demonstrates promising results with an accuracy of 98.91 % and an F1-score of 94.83, showcasing the robustness of the proposed algorithms on a benchmark dataset. The findings hold potential to improve clinical decision-making processes. This study advances medical diagnostics by combining machine learning algorithms with nature-inspired optimization techniques to detect thyroid illnesses in their early stages.

• This article proposes a novel hybrid algorithm that combines the Cuttlefish Optimization Algorithm (CFA) and Simulated Annealing (SA) to find the best features for finding thyroid disease.
• The study uses machine-learning models for classification.
• The integration of machine learning and nature-inspired optimization significantly enhances the diagnostic capabilities of healthcare systems, enabling prompt diagnosis and treatment planning for thyroid disorders.

## Graphical abstract

**Figure:**
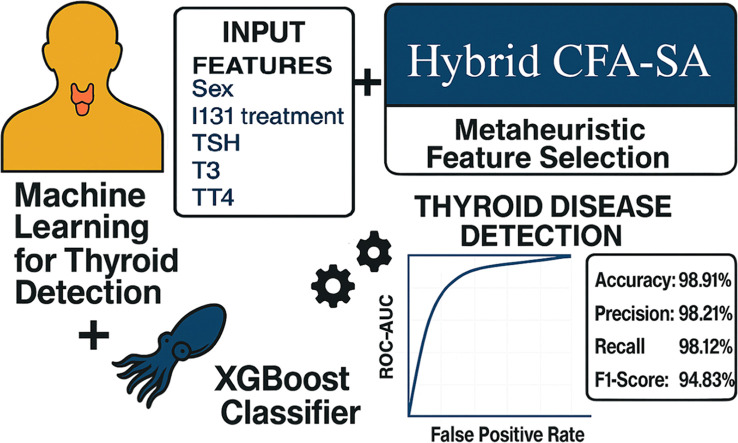

**Specifications table**

**Table:** 

Subject area	Bioinformatics
**More specific subject area**	*Disease detection and computational techniques*
**Name of your method**	Simulated Annealing for Optimal Feature Selection
**Name and reference of original method**	*References are marked*
**Resource availability**	*Specified in Data availability statement*

## Background

The thyroid gland, an endocrine organ, plays a crucial role in the body's metabolism through the secretion of hormones such as triiodothyronine (T3) and thyroxine (T4), which are vital for regulating heart rate, body temperature, and the overall metabolic process, influencing how the body utilizes and stores energy and nutrients. Dysfunction in the thyroid gland can lead to conditions like hyperthyroidism, which is characterised by excessive hormone production, while hypothyroidism is marked by insufficient hormone levels [1]. Both conditions pose significant health risks and can lead to severe disorders if not properly managed.

Hormonal imbalances are not the only conditions that can cause thyroid disorders; the gland can also experience structural problems such as thyroiditis (inflammation), nodules, and multinodular goitre, some of which can lead to malignant tumours. Given the complexity and potential severity of these conditions, an effective and timely diagnosis is essential. The primary treatment for hypothyroidism involves the administration of levothyroxine (Levoxyl), a synthetic hormone replacement. Dosing levothyroxine (Levoxyl) presents significant challenges because it requires tailoring treatment to each patient’s unique needs. Factors such as the patient's thyroid-stimulating hormone (TSH) levels, body weight, and thyroid function influence this customization. Therefore, it is crucial to regularly monitor and adjust therapy to accommodate physiological changes that a patient may experience throughout their lifetime, such as weight fluctuations and hormonal shifts during pregnancy.

Advancements in computational biology and Machine Learning (ML) have significantly impacted the field of medical diagnostics [2], offering tools for more accurate and efficient disease detection. ML algorithms have effectively enhanced the accuracy and reduced the time and cost associated with traditional diagnostic methods in the diagnosis of various conditions, such as heart disease, diabetes, and Parkinson's disease. These algorithms analyse vast amounts of clinical data, identifying patterns and making predictions that support healthcare professionals in their decision-making processes.

Recent studies have explored various machine learning approaches for thyroid disease detection. Decision trees (DT) [[3], [4], [5]], Naive Bayes [6,7], Random Forests (RF) [8], K-nearest Neighbours (KNN) [9,10], and Support Vector Machines (SVM) [11] have all been used successfully by researchers to sort and predict thyroid conditions. However, many of these studies have focused on individual algorithms, which may not fully capture the complexities of thyroid disorders.

## Method details

This paper aims to advance healthcare by enhancing patient outcomes through timely diagnosis of thyroid diseases, leveraging a novel hybrid algorithm. In particular, it tests how the hybrid of the cuttlefish algorithm and simulated annealing performs to find the best features for thyroid disease detection. By using machine learning models like RF, SVM, XG-Boost, and KNN, the research assesses diagnostic performance with measures such as F1-score, recall, accuracy, and precision. The study also explores how integrating machine learning and nature-inspired optimization can significantly improve healthcare systems' diagnostic capabilities. This work intends to serve as a valuable reference for future researchers and healthcare professionals in the domain of medical diagnostics.

In following section literature review provides a comprehensive analysis of the previous investigations. The method details section highlights the operational principles of CFA and SA, as well as the suggested hybrid algorithm for feature selection and classification of thyroid disorders. The study's findings are presented in discussion section with concluding the study.

## Literature review

Sonuç and Salman [12] investigated the effectiveness of various machine learning techniques in classifying thyroid illnesses using patient data from Iraq. They highlighted the exceptional performance of Random Forest in both models, achieving high accuracy rates. The study stressed how important it is to optimise the input attributes and showed that choosing the right features could make some algorithms work better, especially the Multilayer Perceptron and Naïve Bayes methods. These findings illustrate the potential of machine learning to improve thyroid disease classification. They [13] compare how well the machine learning models could diagnose thyroid diseases. The study found that decision trees and random forests consistently outperformed KNN and Naïve Bayes in terms of accuracy. These results provide valuable insights for medical practitioners, illustrating the importance of ensemble techniques like Random Forest in thyroid disease diagnosis.

Begum and Parkavi [14] used data mining techniques to classify thyroid conditions by examining the correlation of TSH, T3, and T4 levels with hyperthyroidism and hypothyroidism across different genders. This study enhances traditional diagnostic methods by leveraging machine learning for improved accuracy. Razia et al. [15] conducted a comparative study using Naïve Bayes, Multiple Linear Regression, and Decision Trees using a UCI dataset. Their research demonstrated the varying efficacy of these algorithms in diagnosing thyroid diseases.

The authors [16] have introduced the Criteria Weighting Based on Confusion Matrix (method) for solving multiclass classification problems. Applied to datasets involving COVID-19, diabetes, and thyroid disease, this approach shows superior performance compared to existing approaches. Kumar et al. [17] analysed a dataset from the UCI repository, applying various machine learning methods to enhance thyroid disease detection and diagnosis. Their study emphasised the critical role of machine learning in thyroid disease prevention. Saleh and Othman [18] conducted a systematic literature review, highlighting the challenges of dealing with imbalanced data for diagnosing thyroid disease. Their meta-analysis identified areas for future research, particularly in addressing data imbalance issues. Srivastava and Kumar [19] optimised a CNN-based model for thyroid nodule classification, achieving significant improvements in accuracy, f-measure, and sensitivity measures compared to other models. This study utilised boundary detection techniques to enhance the model's performance.

In their study [20], Kour et al. created a bagged ensemble model that combines linear discriminant analysis (LDA) classifiers with SMOTE oversampling. This model outperformed conventional machine learning classifiers in thyroid disorder identification, showcasing its potential for clinical application. Brindha and Muthukumaravel [21] compared the efficacy of CNN and SVM algorithms in diagnosing thyroid diseases, finding that CNN achieved superior performance. Dholakia et al. [22] evaluated various machine learning techniques for predicting thyroid disorders in young women. They identified K-Nearest Neighbour (KNN) as the top performer, emphasising the importance of effective classifiers and machine learning algorithms in enhancing diagnostic accuracy. Xing and Bei [23] introduced an enhanced KNN algorithm, addressing traditional limitations in handling large datasets. Their approach incorporated weight assignment, cluster denoising, and density cropping, significantly improving classification efficiency while maintaining accuracy.

## Research gaps

Despite significant advancements in the application of machine learning and deep learning techniques for thyroid disease diagnosis, several critical research gaps remain unaddressed. A close examination of the reviewed studies reveals limitations in methodology, optimization strategies and real-world applicability. Addressing these gaps is essential for developing more robust and interpretable diagnostic systems. The following research limitations have been identified:

• While prior studies have demonstrated competitive performance using classical and ensemble machine learning algorithms for thyroid disease diagnosis, there remains a critical gap in leveraging hybrid metaheuristic-based feature selection techniques.
• Class imbalance is a well-recognized limitation in thyroid disease datasets; however existing frameworks often treat imbalance correction and feature optimization as disjoint processes.
• Despite incremental improvements in predictive accuracy across several studies, very few models have consistently surpassed 97.5 % accuracy on publicly available benchmark datasets.
• Current research predominantly emphasizes algorithmic advancements in classification models while focusing on integration of bio-inspired optimization strategies.

## Method validation

**Fig. 1:**
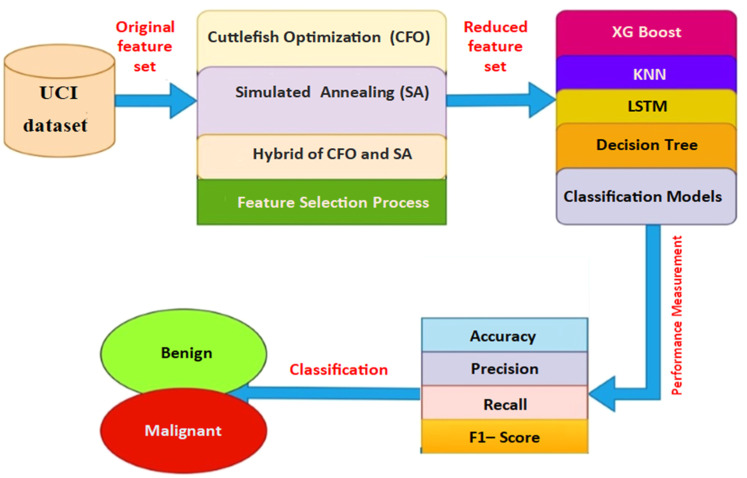
Proposed framework: hybrid cuttlefish optimization and simulated annealing.

This study proposes a hybrid algorithm that refines the feature selection process by combining CFA and SA. The CFA generates initial solutions, while SA further optimizes them by effectively exploring and exploiting the search space. Fig. 1 illustrates the layout of the proposed work.

### The cuttlefish algorithm

CFA algorithm emulates the colour-changing behaviour of the cuttlefish in order to address numerical global optimization challenges. Three distinct cellular layers generate the chromatic and patterned attributes of the cuttlefish through the reflection of light [24].

We employ the CFA as a search method to determine the optimal subset of features. The algorithm emulates the physiological principles employed by cuttlefish in order to change their colors. The algorithm incorporates several cellular layers, such as chromatophores, leucophores, and iridophores. These layers are composed of Chromatophores are specialized cells found beneath cuttlefish's skin that contain pigments and the ability to reflect light. These cells play a crucial role in the modulation and concealment of pigmentation. Iridophores, on the other hand, are pigment cells located beneath the chromatophores. The CFA considers two primary processes: reflection and visibility.

We use reflection to approximate how light reflects in the previously mentioned layers. The concept of visibility refers to the degree of clarity with which the cuttlefish attempts to imitate the patterns that exist in its surroundings. In the context of the cuttlefish algorithm, this represents a variance between the current solution and the optimal one. Eq. (1) describes the computation of the new solution.

(1) P(new)=reflection+visibility

The approach starts by initializing a population of initial solutions, which consists of two distinct subsets of data points: we select one subset and leave the other group unselected. We have selected and saved the optimal subset of points, determined by a higher fitness value.

Eq. (2) represents the initial population generation and distribution over the d dimensions. The top variable stores the optimal solution, while AVtop stores the average of the best solutions obtained.

(2) P[i].points[j]=random*(upperLimit−lowerLimit)+lowerLimit

*Where i =* *1,2,3,4…. N and j* *=* *1,2,3,4……d*

To emulate the six instances of the light reflection mechanism observed in cuttlefish, the population is systematically divided into four distinct groups.

*Group 1 (G1) employs case 1,2*

*Group 2 (G2) utilises case 3,4*

*Group 3 (G3) applies case 5,*

*Group 4 (G4) employs case 6*

The current investigation will consider two distinct cases, namely Case 1 and Case 2, in relation to the G1.

Iridophores cells possess the ability to reflect light emitted by chromatophores cells. To effectively blend with its surroundings, the cuttlefish employs the stretch and shrink mechanisms in chromatophore cells, as well as the reflection of light from iridophore cells. (3), (4), respectively, explain the procedures.

(3) $$reflection_{j}=R*G_{1}\left[i\right]\cdot Points\left[j\right]$$

(4) $$visibility{\;}_{j}=V*BestPoints\left[j\right]-G_{1}\left[i\right].Points\left[j\right]$$

Eqs. (3) and (4) delineate the procedural steps described in the problem statement. In this context, "G1″ denotes a collection of chromatophore cells used to simulate scenarios 1 and 2. The index "i" identifies each cell within this group, while "Points[j]" denotes the j-th point.

The variable "R" represents the degree of reflection, Meanwhile, the variable "V" indicates the pattern's visual acuity. We compute the values of R and V using specific formulas derived from the described calculations.

(5) $$R=random\left(\right)*\left(r_{1}-r_{2}\right)\;+r_{2}$$

(6) $$V=random\left(\right)*\left(v_{1}-v_{2}\right)\;+v_{2}$$

Where, Random () is a mathematical function that generates random numbers in the range (0, 1). We use the constants r1 and r2 to calculate the time interval between chromatophore cell stretches. For the final pattern observation, we use the values v1 and v2 as constants to determine the range of visibility angles. In a few cases, we assign the value of V and R to 1, whereas in others, we compute it. In this specific group, we fix the value of V at 1 and calculate the value of R.

The present analysis will consider cases 3 and 4 in relation to G2.

The iridophores cells function as a mirror, reflecting incoming light in a specific hue. To cover up organs or aid in concealment, we employ cells called iridophores. We select the optimal solution to represent the hidden organs. Therefore, we leave the method for determining visibility unchanged and revise the formula for determining reflection.

(7) $$reflection_{j}=\;R*Best.Point\left[j\right]$$

Where R is assigned as value 1 for this group, and V is to be calculated.

The fifth case study pertaining to the G3.

In our study, we assumed that the incoming color represents the optimal choice, while the reflected color may vary within a range close to this optimal value. This approach accounts for the resemblance between the incoming and reflected colors. The visibility parameter defines the range around the best, establishing the interval within which the reflected color may fall.

Visibility establishes the optimal interval. We update Eqs. (3) and (4) to determine visibility and reflection as follows:

(8) $$reflection_{j}=\;R*Best.Point\left[j\right]$$

(9) $$visibility{\;}_{j}=V*\left(Best.Points\left[j\right]-AV_{top}\right)$$

where AV_top_ represents the average value of Best points. R is assigned the value 1 and the value of V is being computed.

The sixth case study pertaining to the G4.

In this scenario, cuttlefish can adapt to their surroundings by utilizing any color that enters the environment. Leucophore cells then reflect this color back, representing it as a random solution in a simulation.

### Simulated annealing

SA [25] starts with an initial set of solutions at a high temperature. At each iteration, the temperature decreases gradually. In each iteration, the algorithm generates a new solution by perturbing the current solution. A probability function determines the acceptance of this new solution by accounted for the difference in objective function values and the current temperature.

During the initial high-temperature phase, the algorithm explores a broader solution space by accepting solutions that may even worsen the objective function value. When the temperature increases, the algorithm's propensity to accept suboptimal solutions escalates due to the probabilistic computations involved. This mechanism promotes a more extensive exploration of the search space.

As the temperature decreases, the algorithm's selectivity increases, progressively prioritising solutions that enhance the objective function value. This shift fosters exploitation, suggesting that when the algorithm is within the correct search space, it is no longer necessary to explore other regions of the search space. Instead, the algorithm should focus on converging towards identifying the global maximum.

The mathematical equations used in the SA algorithm are as mentioned. You can gradually lower the temperature using the following Eq. (10)

(10) t=t*α

Here, t represents the temperature, and α represents the cooling rate.

The Eq.. (11) for the acceptable probability at which we can accept the solution is represented as follows:

(11) $$P=\{1\;if\Delta c\leq 0\;e^{\frac{\Delta c}{t}}\;if\Delta c>0$$

where, delta c representing the cost change and t denoting the current temperature for the generating neighbour, we can formulate the following Eq. (12).

(12) Neighbour=current+random(−0.1,.1)

**Hybrid approach** The hybrid approach combines CFA with SA to refine the feature selection process. The CFA generates initial solutions and refines them, while SA further optimizes the solutions by exploring and exploiting the search space effectively. The hyper parameters values of the proposed algorithm are given in Table 1.

**Table:** Algorithm (Proposed).

1.	Determine the population size (N) & Initialize population, set initial value of r1, r2, v1, v2
2.	Evaluate the fitness using the objective function and keep best solution in top
3.	Divide Population in four groups (S1, S2, S3, S4).
4.	While iter ≤ MaxIter:
5.	compute average of best solutions as AVtop
6.	Case (1,2)
	for S1 (*i* = 1,2,3…… Size_of_S1)
	Generate solution by using Eq (3,4,5,6)
7	Case (3,4)
7.1	for S2 (*i* = 1,2,3…… Size_of_S2)
	Generate solution by using Eq (7)
7.2	Set the intial parameter of SA
	SA loop for solution using Eq (10,12)
8	Take better solution obtained from 6.1 & 6.2
9	Case (5)
9.1	for S3 (*i* = 1,2,3…… Size_of_S3)
	Generate solution by using Eq (8,9)
9.2	SA loop for solution using Eq (10,12)
10	Take better solution obtained from 8.1 & 8.2
11.	Case (6)
	for S4 (*i* = 1,2,3…… Size_of_S4)
	Generate any random solution using Eq (2)
12	Return the Optimal solution

**Table 1:** Hyper parameters.

Hyper parameter	values
r1	.5
r2	−2
v1	1
v2	−1
initial_temperature	9
cooling_rate	.95
num_iterations	100

### Dataset

**Fig. 2:**
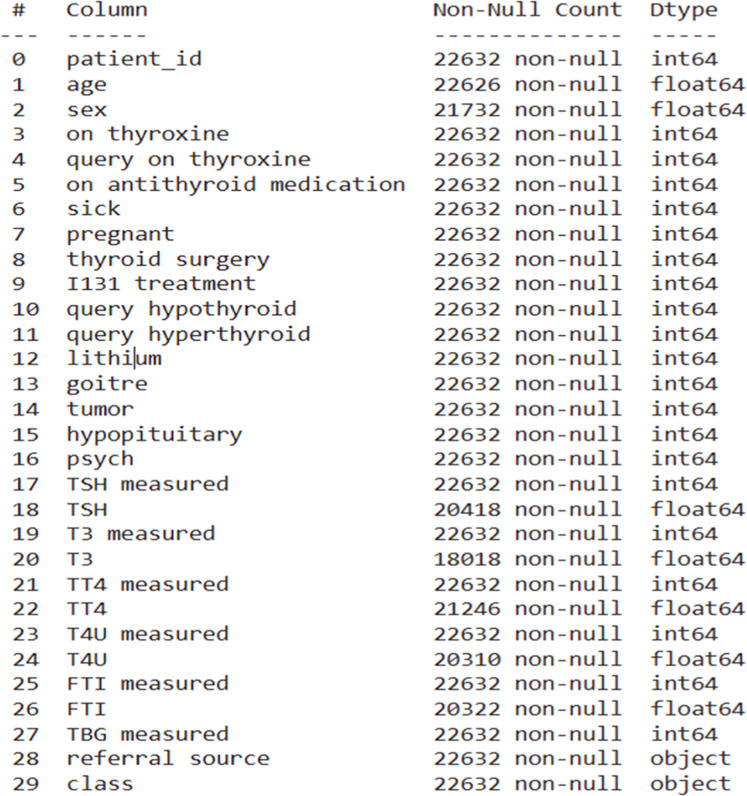
UCI Thyroid dataset attibutes.

We obtained the benchmark dataset for our study from the UCI Machine Learning Repository, which specifically focused on thyroid disease datasets. The dataset we used consists of 22,632 sample observations, each of which has 28 unique attributes. Fig. 2 shows the attributes of UCI thyroid dataset.

## Discussion

**Fig. 3:**
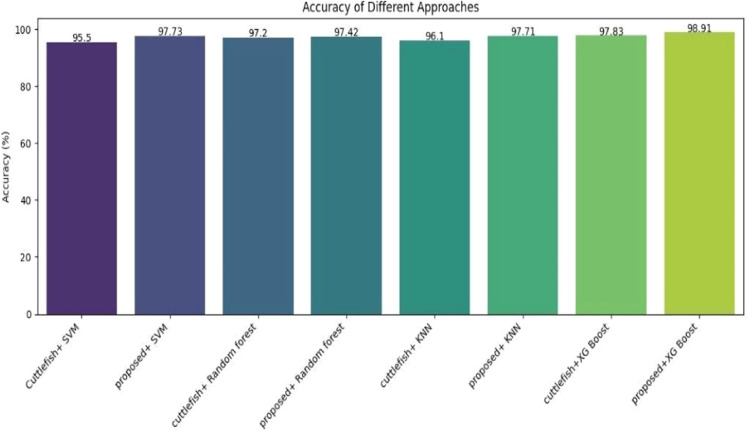
Comparison of accuracy of implemented approaches.

We compared the accuracy, precision, recall, and F1 score of different classification models with the Cuttlefish optimization algorithm and the proposed optimization approach shown in Table 2. A graphic representation of the accuracy obtained by implementing different approaches is depicted in Fig. 3. The proposed algorithm extracts 7 features in total: "age," "sex," "I131 treatment," "TSH," "T3," "TT4", and "T4U."

**Table 2:** Brief evaluation of implemented scenario.

APPROACH	Accuracy	Precision	Recall	F1 score
Cuttlefish+ SVM	95.5	93	92.5	90.75
Cuttlefish+ Random forest	97.2	94.5	93.8	92.15
cuttlefish+ KNN	96.1	93.2	93	91.1
Cuttlefish+XGBoost	97.83	95.2	94.8	93
proposed+ SVM	97.73	96.5	96	93.5
proposed+ Random forest	97.42	97	96.12	93.99
proposed+ KNN	97.71	96	95.51	92.37
proposed+XGBoost	98.91	98	98.12	94.83

**Fig. 4:**
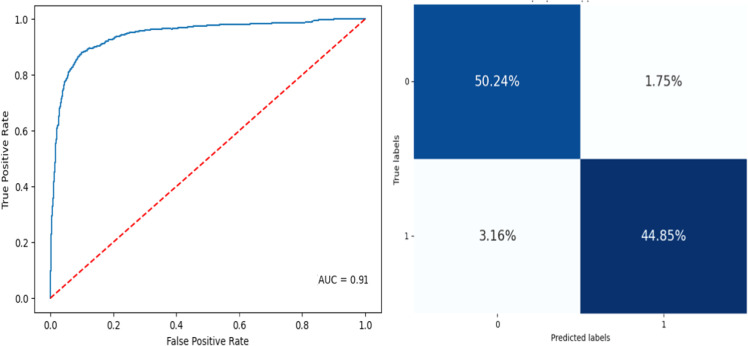
ROC-AUC curve and confusion matrix of CFA+XGBoost.

**Fig. 5:**
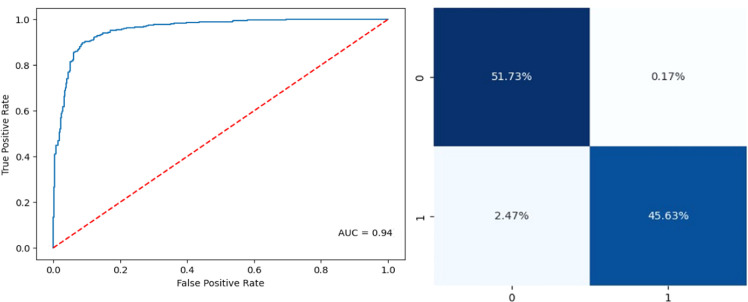
ROC-AUC curve and confusion matrix of proposed+XGBoost.

The proposed approach, when combined with XGBoost, demonstrated the highest overall performance among the models, achieving accuracy of 98.91 %, precision of 98 %, recall of 98.12 %, and an F1 score of 94.83. This superior performance suggests that the proposed method significantly enhances the model's ability to correctly identify true positives and reduce false positives and negatives, making it highly effective for classification tasks. Fig. 4–5 depict the metrics of the confusion matrix, and the AUC-ROC for CFA-SA algorithm. Compared to the CFA, the proposed method showed consistent improvements.

## Comparison with prior studies

The proposed approach achieves an accuracy of 98.91 %, precision of 98.00 %, recall of 98.12 %, and an F1 score of 94.83, outperforming several existing methods, Results of effective approaches is shown in Table 3 . Compared to the Adaptive Elephant Herd Optimization Algorithm (AEHOA) integrated with a CNN which achieved an accuracy of 88.25 % and an F1 score of 87.67, the proposed method shows significant improvement [26]. Similarly, the Vgg-19-LSTM model, combining LSTM-CNN with hybrid optimization, achieved an accuracy of 98.8 % and an F1 score of 93.24, slightly less balanced than the proposed method [27]. For early detection of thyroid disorders, the study evaluated Logistic Regression, SVM, and DT models, identifying the highest accuracy at 98.00 %, but the proposed method still surpasses this with better overall precision and recall [28]. Lastly, the comparison with Naive Bayes, Logistic Regression, KStar, and J48 classifiers indicates that the J48 model, which achieved 97.80 % accuracy, falls short of the proposed approach’s performance [29].

**Table 3:** Summary of comparison with prior studies.

APPROACH	Accuracy	Precision	Recall	F1 score
Jopate, Rachappa, et al. [26]	88.25	91.42	NA	87.67
Mohan, E., et al. [27]	98.8	99.2	96.56	93.24
Akash, K.Thirumala, et al. [28].	98.00	99.00	98.00	99.00
Saini, Archana, et al. [29]	97.81	-	-	-

## Conclusion

Over the past decade, thyroid disease (TD) has emerged as a pressing health concern, with a notable impact on morbidity and mortality rates. Among endocrine disorders, thyroid disease ranks as one of the most prevalent conditions worldwide, particularly affecting women. The proposed hybrid approach, integrating the CFA with SA for optimal feature selection, demonstrates promising results in thyroid disease detection. The experimental results show high accuracy and F1-score, indicating that the approach is effective. This study contributes to the advancement of medical diagnostics by leveraging nature-inspired optimization techniques and machine learning methodologies. Future research can explore the application of the proposed hybrid approach to other medical diagnostic problems.

## Limitations

Lack of Explainable AI methods reduces the interpretability of model decisions, which is important for clinical trial. The model might ignore comorbid effects since it does not distinguish thyroid dysfunction patterns between diabetic and non-diabetic patients. Using stationary datasets limits understanding of temporal fluctuations and disease development. There is Lack of external validation across different populations reduces the generalizability of the findings and class imbalance in the dataset may distort predictions towards more common thyroid disorders. Further studies can utilize more datasets based on real time data collection to detect thyroid disease.

## Ethics statements

Not Applicable.

## CRediT author statement

Kapil Shrivastava: Conceptualization, original draft preparation,Saroj Pandey: Writing – Reviewing and Editing,Mayank Namdev Methodology, Rishav Dubey: Software Development and Analysis, Vipin Tiwari: Method validation, original draft preparation and funding acquisition; Aditi Sharma: Formal analysis and writing.

## Declaration of competing interest

The authors declare that they have no known competing financial interests or personal relationships that could have appeared to influence the work reported in this paper.

## Data Availability

Data will be made available on request.
